# 4E-BP1 as an oncotarget

**DOI:** 10.18632/aging.100794

**Published:** 2015-08-16

**Authors:** Qing-Bai She

**Affiliations:** Markey Cancer Center, University of Kentucky, Lexington, KY, USA

The cap-dependent mRNA translation process that enables post-transcriptional control of gene expression and protein synthesis is tightly regulated in eukaryotes. Deregulation of this process, particularly in the formation of the eIF4F translation initiation complex—comprising the eIF4E mRNA cap-binding protein, the eIF4G scaffolding protein and the eIF4A RNA helicase—is associated with cancer development and progression. It is widely believed that translation of certain key oncogenic mRNAs is strongly dependent on the level of eIF4E. Consequently, expression of these oncogenic mRNAs is preferentially and dis-proportionately affected by eIF4E availability and is sensitive to alteration of its level. Free eIF4E level can be increased substantially in cancer cells by a number of mechanisms, including elevated eIF4E expression, decreased expression of eIF4E inhibitory binding proteins, 4E-BPs, and release of eIF4E from 4E-BPs by inactivating phosphorylation of 4E-BPs results from the oncogenic activation of PI3K/AKT and RAS/RAF/MEK/ERK signaling pathways.

Our recent studies have revealed that 4E-BP1 is a critical regulatory node that integrates oncogenic signals of the P3K/AKT and RAS/RAF/MEK/ERK pathways for cancer progression and metastasis [1-3]. Mutations in genes that encode components of these two pathways occur at high frequency in cancer. Moreover, the AKT and ERK pathways are often concurrently activated by separate mutations in many human tumors. For instance, *KRAS* and *PIK3CA* mutation occur simultaneously in colorectal carcinoma; *BRAF* and *PIK3CA* mutation in thyroid carcinoma; and *BRAF* and *PTEN* mutation occur simultaneously in melanoma. A number of small molecule inhibitors targeting components of these two pathways have been tested for the treatment of cancer in preclinical and clinical studies, but have shown only limited activity as a single agent. Our work demonstrated that in tumors such as colorectal cancer (CRC) with coexistent activation of both PI3K/AKT and RAS/ERK pathways, drug resistance, following inhibition of either pathway, is associated with redundant activation of eIF4E-initiated cap-dependent translation through convergent phosphorylation of the translational repressor 4E-BP1 mediated by the AKT and ERK pathways. We found that combined inhibition of both pathways is required to maximally inhibit 4E-BP1 phosphorylation and subsequent induction of 4E-BP1 binding to eIF4E to prevent cap-dependent translation of oncogenic mRNAs (e.g. survivin) and ultimately achieve antitumor activity. Furthermore, we showed that a genetically-modified non-phosphorylated 4E-BP1 mutant that binds constitutively to eIF4E and represses eIF4E-dependent oncogene expression exerts similar inhibitory effects on tumor growth and metastasis as the combined pharmacological inhibition of AKT and ERK pathways, whereas loss of 4E-BP1 expression induces epithelial-mesenchymal transition through translational control of snail expression and its activity to promote metastatic progression of CRC and reduce CRC dependence on AKT/ERK signaling for translation, survival and motility. Collectively, these findings support the conclusion that deregulation of 4E-BP1 phosphorylation-mediated assembly of eIF4F translation initiation complex causes CRC resistance to its upstream kinase targeted therapies and thus maintain the transformed phenotype for CRC progression and metastasis. Of notice, several lines of evidence have also established incomplete inhibition of 4E-BP1 phosphorylation, amplification of eIF4E or increased eIF4E/4E-BP ratio as a mechanism of primary resistance to anti-PI3K/AKT/mTOR and anti-BRAF/MEK/ERK pathway inhibitors in a variety of cancers [4-6].

Our data and others strongly suggest that 4E-BP1 phosphorylation is an important biomarker and therapeutic target in cancer. It is well know that mTOR kinase complex 1 (mTORC1) phosphorylates 4E-BP1, which in turn, activates cap-dependent translation and promotes protein synthesis. Our recent studies demonstrated that the effects of AKT and ERK activation on translational regulation of CRC progression are largely mediated by mTORC1 [2], suggesting that targeting mTOR is an attractive strategy for cancer therapy. Nevertheless, incomplete inhibition of 4E-BP1 phosphorylation and a concomitant activation of AKT via loss of a negative feedback mechanism by mTOR inhibitors, including rapalogs and mTOR kinase inhibitors, have been implicated in reduction of their therapeutic efficacy. Our most recent findings revealed a critical role of PRAS40, a mTORC1 inhibitor, as a key integrator of mTORC1 and AKT signaling for 4E-BP1-mediated translational regulation of tumor progression, and indicated that the redundant phosphorylation of PRAS40 on Ser183 and Thr246 sites by both mTORC1 and AKT signaling is a novel mechanistic basis for the acquired resistance to mTOR inhibitors in cancer cells [7].

Our findings further suggest that directly targeting the convergence of multiple oncogenic signals on eIF4F translation initiation complex may provide a promising strategy for improving cancer therapy. Several translation initiation inhibitors, including eIF4E antisense-oligonucleotides and the eIF4A inhibitor silvestrol, have recently produced encouraging anti-tumor effects with limited toxicity. Future works require additional clinical correlative studies and await the development of clinically effective translation initiation inhibitors for cancer treatment, either as single agents or as part of combination therapy.
